# Impact of using different predictive equations on the prevalence of chronic byssinosis in textile workers in Pakistan

**DOI:** 10.1136/oemed-2021-107680

**Published:** 2021-11-19

**Authors:** Asaad Ahmed Nafees, Muhammad Zia Muneer, Sara De Matteis, Andre FS Amaral, Peter Burney, Paul Cullinan

**Affiliations:** 1 Department of Community Health Sciences, Aga Khan University, Karachi Address: Stadium Road, P.O. Box-3500, Karachi (74800), Pakistan. Tel (off.): + 92 21 3486 4884, Fax: + 92 21 3493 2095; 2 Population Health and Occupational Disease, National Heart and Lung Institute (NHLI), Imperial College London, London, UK; 3 Department of Community Health Sciences, Aga Khan University, Karachi; 4 Population Health and Occupational Disease, National Heart and Lung Institute, Imperial College London, London, UK; 5 Population Health and Occupational Disease, National Heart and Lung Institute, Imperial College London, London, UK; 6 Population Health and Occupational Disease, National Heart and Lung Institute, Imperial College London, London, UK; 7 Population Health and Occupational Disease, National Heart and Lung Institute, Imperial College London, London, UK

## Abstract

**Objective:**

byssinosis remains a significant problem among textile workers in low- and middle-income countries. Here we share our experience of using different prediction equations for assessing ‘chronic’ byssinosis according to the standard WHO classification using measurements of FEV_1_.

**Methods:**

we enrolled 1910 workers in a randomized controlled trial of an intervention to improve the health of textile workers in Pakistan. We included in analyses the 1724 (90%) men who performed pre-bronchodilator spirometry tests of acceptable quality. We compared four different equations for deriving lung function percentage predicted values among those with symptoms-based byssinosis: the third US National Health and Nutrition Examination Survey (NHANES-III, with “N. Indian and Pakistani” conversion factor); the Global Lung Function Initiative (GLI, “other or mixed ethnicities”); a recent equation derived from survey of a western Indian population; and one based on an older and smaller survey of Karachi residents.

**Results:**

58 men (3.4%) had symptoms-based byssinosis according to WHO criteria. Of these, the proportions with a reduced FEV_1_ (< 80% predicted) identified using NHANES, GLI, Indian and Pakistani reference equations were 40%, 41%, 14% and 12%, respectively. Much of this variation was eliminated when we substituted FEV1/FVC ratio (<LLN) as a measure of airway obstruction.

**Conclusion:**

accurate measures of occupational disease frequency and distribution require approaches that are both standardised and meaningful. We should reconsider the WHO definition of ‘chronic’ byssinosis based on changes in FEV_1_, and instead use the FEV_1_/FVC.

## Introduction

Byssinosis remains a significant problem among textile workers in low and middle-income countries where much global production is now located (1, 2). The disease is characterized by work-related symptoms of chest tightness and dyspnoea and, in its chronic form, by reductions in FEV_1_. There are two classification systems currently in place for byssinosis: that produced by the World Health Organisation (WHO) and the ‘Schilling criteria’ (3, 4). The most severe grade in the latter includes decrements in FEV_1_ in addition to presence of symptoms. The WHO approach, in contrast, recommends that respiratory symptoms and chronic changes in lung function are considered ‘together’, albeit as distinct health outcomes in epidemiological surveys, and that measurements of FEV_1_ should be compared with ‘data obtained from local populations or similar ethnic and social class groups’ (3). Such data, however, are seldom readily available. Here we share our experience of using different prediction equations for assessing ‘chronic’ byssinosis in Pakistani textile workers.

## Methods

We recently completed the baseline survey of a cluster randomized trial of a multifaceted intervention to reduce the incidence of byssinosis among textile manufacturers in Karachi, Pakistan (5). We enrolled 1910 workers from 38 textile mills. Following ERS guidelines, trained technicians undertook pre- and post-bronchodilator spirometry using EasyOne spirometers (ndd Medizintechnik AG) and recorded up to eight measurements of FEV_1_, FVC and their ratio (6). We reviewed all spirograms; the analyses below include the 1724 (90%) men who performed pre-bronchodilator spirometry tests of acceptable quality.

We compared four different equations for deriving lung function: those established through the third US National Health and Nutrition Examination Survey (NHANES-III; “Caucasian”) (7) with a conversion factor of 0.9 recommended for N. Indian and Pakistani individuals (8); the Global Lung Function Initiative (GLI, “other or mixed ethnicities”) equations (9); a recent equation derived from survey of a western Indian population (n=1258) aged 19-88 years (10); and one based on an older and smaller (n=504) survey of Karachi residents aged 16-65 years (11). We classified workers using the WHO recommended FEV_1_ cut-off for identification of workers at risk of developing permanent pulmonary impairment: FEV_1_ <80% predicted. We compared results based on this classification with one where we replaced FEV_1_ by FEV_1_/FVC ratio considering values below the normal limit of normality (LLN) to be abnormal. We undertook analyses in Microsoft Excel.

The study was approved by the ethics committees at Aga Khan University, Karachi (2019-0962-3710), the National Bioethics Committee in Pakistan (4-87/NBC-402/19/483), and Imperial College London (19IC4968).

## Results

Using symptom classification (alone) the prevalence of byssinosis was 3.4% (n=58) and 3.9% (n=67) according to WHO and Schilling’s criteria, respectively – reflecting the grade ½ (9 workers) in the latter. Of the 58 men with byssinosis according to WHO criteria, the proportion with a reduced FEV_1_ (<80% predicted) varied according to which set of predictive equations was used, from 40%-41% with those from NHANES and GLI, to 12%-14% with the more locally derived models (Table 1). Much of this variation was eliminated when we substituted FEV_1_/FVC ratio (<LLN) as a measure of airway obstruction; in particular, the estimates derived from using the GLI, and Indian reference equations were very similar. We observed the same patterns when estimating the prevalence of airway obstruction in the total mill population.

## Discussion

The substantial variation in the prevalence of abnormal FEV_1_, consistent with standard classifications of ‘chronic’ byssinosis, resulting from the use of different lung function prediction equations in this population reinforces the WHO recommendation that reference data from ‘local’ populations be used. Whether the stipulation for data from ‘similar social class groups’ is met is more difficult to ascertain since these are seldom reported for the populations from which predicted values are derived. It is evident that the widely used GLI (in which there is a lack of representation of South Asian populations) and NHANES III equations (even after adjustment) give a very different picture of the prevalence of lung function abnormality when this is expressed by FEV_1_, and therefore may not be useful in a local/regional South Asian context. Much textile manufacture now takes place in populations that are poorly served by spirometric norms, and the problem we have identified in Pakistan will be reflected in many other LMICs (1, 2). In contrast, substitution of FEV_1_ with FEV_1_/FVC ratio, with a LLN criterion of abnormality, produces estimates of airway obstruction that are relatively stable across different prediction equations, including the GLI. Since the FEV_1_ is correlated with the FVC, it is not an unambiguous measure of obstruction, and for this the FEV_1_/FVC, in which the FEV_1_ is adjusted for lung size, is preferable for defining obstruction. In the current context this has the added advantage that it is largely independent of ethnicity, the ethnic differences in FEV_1_ and FVC largely cancelling each other out.

A potential limitation of our work includes the effect of a ‘healthy worker effect’ and the consequent underestimation of the risk of byssinosis in this context; it is improbable that this will have affected our findings in relation to the relative merits of FEV_1_ and FEV_1_/FVC ratio. Reproducible measurements of FVC are more difficult than those of FEV_1_ and require greater technical skill. Finally, the information provided by the authors of the reference equation for Pakistanis (11) was insufficient to calculate an LLN.

Notwithstanding this, all efforts should be made to reduce exposures to cotton dust to identify byssinosis early through periodic workplace surveillance for the presence, nature, and extent of characteristic symptoms and before lung function loss has occurred.

Exposures in the workplace may be important causes of respiratory disease (12) and especially so in LMICs where occupational health and safety measures may be poor. The basis for the prevention of the important public health burden of occupational disease is accurate measures of its frequency and distributions which requires approaches to its recognition and classification that are both standardised and meaningful. We should reconsider the WHO definition of ‘chronic’ byssinosis based on changes in FEV_1_ and substitute it with the use of FEV_1_/FVC.

## Figures and Tables

**Table 1:** proportion of workers with airway obstruction, by different criteria and reference equations, in those with symptoms of byssinosis and in the total mill population

Metric of airflow obstruction	Workers with symptoms of byssinosis (n=58)				Total mill population (n=1724)			
	NHANES (7)	GLI (9)	Indian (10)	Pakistani (11)	NHANES	GLI	Indian	Pakistani
FEV_1_<80% predicted	40%	41%	14%	12%	1.3%	1.4%	0.5%	0.4%
FEV_1_/FVC ratio<LLN	12%	24%	21%	NA	0.4%	0.8%	0.7%	NA

NHANES: National Health and Nutrition Examination Survey III

GLI: Global Lung Initiative

NA: not available

## Data Availability

data are available on reasonable request.

## What this paper adds

### What is already known about this subject?

Byssinosis is a significant problem among textile workers in low and middle-income countries. It is primarily identified by its characteristic symptoms, but more severe, chronic disease is accompanied by airway obstruction. The current, WHO classification suggests that obstruction is measured through FEV_1_.

### What are the new findings?

In the course of a large trial of a workplace intervention in Pakistani textile mills, the use of different prediction equations for measuring reductions in FEV_1_ to widely varying estimates for the prevalence of chronic byssinosis. Substitution of FEV_1_ by FEV_1_/FVC substantially reduced the variation.

### How might this impact on policy or clinical practice in the foreseeable future?

Since accurate measures of occupational disease frequency and distribution require approaches that are both standardised and meaningful, we should reconsider the WHO definition of ‘chronic’ byssinosis based on changes in FEV_1_ and replace it with FEV_1_/FVC.
